# Catching more flies with vinegar

**DOI:** 10.7554/eLife.10535

**Published:** 2015-09-09

**Authors:** Genevieve C Jouandet, Marco Gallio

**Affiliations:** Department of Neurobiology, Northwestern University, Evanston, United States; Department of Neurobiology, Northwestern University, Evanston, United Statesmarco@northwestern.edu

**Keywords:** olfaction, sNPF, insulin, RNA-seq, two-photon imaging, Tachykinin, *D. melanogaster*

## Abstract

Two signalling pathways work together to reshape olfactory responses so that hungry flies are attracted to food sources they would otherwise ignore.

**Related research article** Ko KI, Root CM, Lindsay SA, Zaninovich OA, Shepherd AK, Wasserman SA, Kim SM, Wang JW. 2015. Starvation promotes concerted modulation of appetitive olfactory behavior via parallel neuromodulatory circuits. *eLife* **4**:e08298. doi: 10.7554/eLife.08298

**Image** Hunger changes how the fly olfactory system processes food odors

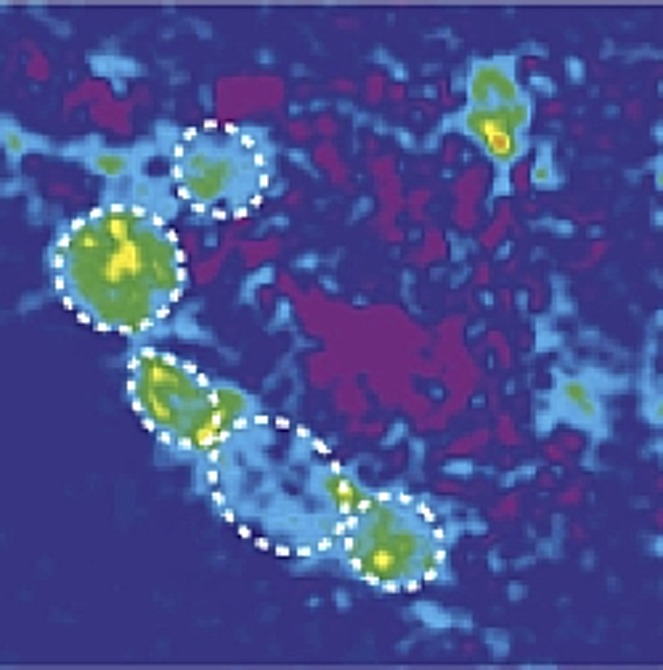

A common expression would have us believe that ‘*you can catch more flies with honey than with vinegar*’. But this is not true in the case of the fruit fly *Drosophila melanogaster* (xkcd, 2007). Adult flies forage for microbes on overripe fruit, relying on their sense of smell to detect the acetic acid (the chemical that gives vinegar its pungent aroma) that accumulates as the fruit ferments. However, flies tend to ignore or even avoid both low levels of vinegar (which suggest that the fruit is not ripe enough) and high levels of vinegar (which suggest that the fruit might be rotten).

Now, in *eLife*, Jing Wang and co-workers at the University of California, San Diego—including Kang Ko as first author—elegantly reveal what happens in flies' brains that allows them to pursue a broader range of vinegar odor concentrations when hungry (Ko et al., 2015). Their data also show that starvation has a more nuanced influence on the early processing of olfactory information than was previously anticipated: hunger does more than just tune up the flies' sensitivity to food odors. Instead, it triggers specific responses (both excitatory and inhibitory) that encourage the flies to forage on sub-optimal food sources. In doing so, Ko et al. possibly provide additional evidence to support the notion that it is not wise to go grocery shopping on an empty stomach, lest hunger signals may impair your ability to discriminate good food from bad.

Ko et al.'s work is the culmination of a series of studies that have addressed how *Drosophila* process information about this important food odor. In fruit flies, much like in humans and other vertebrates, the olfactory neurons that detect specific volatile chemicals wire up to discrete clusters of synapses within the brain called glomeruli. Olfactory neurons that detect the same chemical all connect to the same glomerulus. Depending on the concentration, vinegar odor activates 6 to 8 of the 40 or so glomeruli in the fruit fly brain. However, a previous landmark study from the Wang group revealed that the activity of a single olfactory glomerulus, referred to as DM1, could explain most of a fly's attraction to vinegar (Semmelhack and Wang, 2009). Turning off the receptors that connect to DM1 caused the flies to ignore the odor of vinegar. On the other hand, restoring only the activity of DM1 neurons in otherwise ‘anosmic’ flies (that is, flies that have lost almost all sense of smell) was enough to make them attracted to vinegar again.

Higher concentrations of vinegar recruit just one extra glomerulus, called DM5, and the activity of DM5 on its own can explain why flies avoid vinegar if the odor is too strong (Semmelhack and Wang, 2009). Hence, the competitive interaction between DM1 and DM5 (which are activated at different vinegar odor concentrations) may ultimately determine whether the fly decides to approach a potential food source or to stay away.

Hunger has a profound impact on animal behavior, and hungry flies find a small drop of vinegar-laced food much more quickly than flies that have been fed (Root et al., 2011). The hormone insulin indirectly mediates this effect. Starvation causes insulin levels to plummet, triggering a chain of events that ultimately causes DM1 olfactory neurons to increase the expression of a specific receptor protein. This receptor detects a signaling molecule called ‘short neuropeptide F’. Upon binding to the receptor, this neuropeptide effectively amplifies, or turns up the ‘gain’ of, DM1 activity. Since DM1 neurons control a fruit fly's attraction to vinegar, this finding seemed to elegantly explain how insulin signaling can lead hungry flies to look more widely for food.

It now transpires that this is not the whole story. By extending the range of odor concentrations tested, Ko et al. now find that this mechanism only explains how hungry flies boost their attraction to low vinegar odor concentrations. At higher concentrations, starved flies still pursue vinegary food more robustly than fed controls, even when signaling mediated by short neuropeptide F is reduced (Ko et al., 2015). Could an additional neuropeptide account for this difference? To search for this missing hunger signal, Ko et al. surveyed other receptor proteins, looking for those that were increased in sensory neurons as a result of starvation. The Tachykinin receptor (called DTKR for short) emerged as a strong candidate, especially because it was known that it can tune down the responses of the fly's olfactory neurons (Ignell et al., 2009).

The rest of Ko et al.'s story beautifully follows a logical script: knocking down the levels of DTKR indeed reduced food-finding behavior in hungry flies exposed to high, but not low, vinegar odor concentrations. Similarly, DM5 (the glomerulus responsible for avoidance of high levels of vinegar) was less active in starved flies, but its activity could be brought back up to that of a fed fly when DTKR was knocked-down. Finally, Ko et al. identified insulin as the likely signal that acts upstream of DTKR in starving flies.

Taken together, the data suggest a model in which falling insulin levels in starving flies trigger two complementary neuropeptide signaling systems involving short neuropeptide F and Tachykinin. One helps the transmission of signals at the DM1 glomerulus, which makes the flies more sensitive to attractive food odors. In parallel, the other turns down transmission at DM5, which makes the flies less likely to avoid normally unpleasant or aversive smells. Together, these systems allow flies to pursue less-than-optimal food sources in times of shortage (Figure 1).

**Figure 1.**
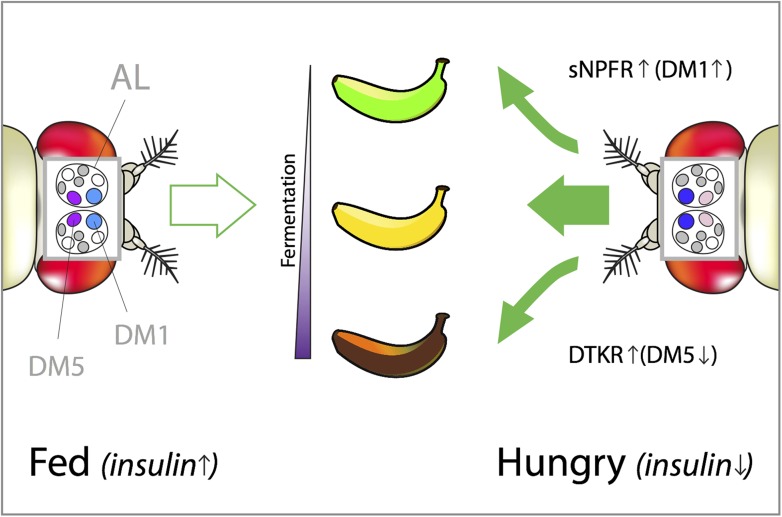
How hunger influences the attractiveness of food odors in *Drosophila*. Vinegar (or acetic acid) is the ultimate product of the fermentation process in fruit, which is why fruit flies are attracted to vinegar odor. However, both low and high concentrations of vinegar odor leave flies indifferent (left). This is because low concentrations indicate that the fruit is just-ripe (green banana), whereas high concentrations mean that it is rotten (brown banana). Hungry flies behave differently because the low levels of insulin caused by starvation trigger two distinct neuropeptide signaling systems that reshape their olfactory responses (right). In hungry flies, the receptor for short neuropeptide F (called sNPFR) is upregulated in a subset of olfactory neurons. This helps the transmission of signals within the DM1 glomerulus, which increases the sensitivity to low concentrations of attractive food odors. In parallel, elevated Tachykinin signaling (through the DTKR receptor) inhibits the transmission of signals within the DM5 glomerulus. This decreases the avoidance of normally unpleasant smells (such as high concentrations of vinegar). Together these effects allow the pursuit of less-than-optimal food sources (depicted by the green arrows pointing toward the just-ripe and rotten bananas). DM1 and DM5 are specific glomeruli found in the antennal lobe (AL) of the fly brain and their color intensity represents the strength of their activation in fed vs hungry flies.

This study powerfully demonstrates the strengths of the fly model as a platform to study how the brain computes sensory stimuli. From clever behavioral assays, to sophisticated genetic manipulations and imaging of brain activity, the work describes how an important sensory cue is handled in different ways depending on the internal state of the animal (that is, hungry or not). Since what is true for the fly is often—at least in outline—true for man, the area of research is now ripe to contribute principles of sensory processing that may be applicable to many, if not all, animal species.
